# Radiographic measurements of the hoof in generally sound donkeys with emphasis on the front limbs

**DOI:** 10.3389/fvets.2024.1505253

**Published:** 2024-12-11

**Authors:** Juliana Wacker, Kyra Schaus, Anabell Jandowsky, Kathrin Büttner, Michael Röcken, Claus Peter Bartmann

**Affiliations:** ^1^Clinic for Equine Surgery and Orthopedics, Justus-Liebig-University Giessen, Giessen, Germany; ^2^Arche Warder – Zentrum für alte Haus-und Nutztierrassen e.V, Warder, Germany; ^3^Unit for Biomathematics and Data Processing, Justus-Liebig-University Giessen, Giessen, Germany

**Keywords:** equid, donkey, foot, hoof, forelimb, X-ray, radiometry, measurements

## Abstract

**Introduction:**

Due to the tremendous importance of the hoof for the welfare of equids, plenty of studies have investigated the foot conformation of horses, which can be objectively described by morphometric measurements. Although the foot conformations of horses and donkeys differ, only poor data exists describing the physiological foot conformation of donkeys. The objective of this study was to provide reference values for different radiographic parameters for healthy donkeys. Additionally, this study aimed to evaluate differences between the left and right limb or between the medial and lateral parts of the hoof and the dependence of various parameters on size, age, and weight.

**Materials and methods:**

Lateromedial and dorsopalmar radiographs were taken of 46 generally sound donkeys, that were lame-free in walk, following a hoof correction due to their hoof pastern axis. With the help of radiopaque markers, several measurements were performed on the radiographs. The statistical analysis was carried out by determining mean values and standard deviations. All parameters were tested for significant differences between the left and right limbs as well as the medial and lateral hoof parts. The methodology included using a paired T-test after proof of a normal distribution of the data using a Shapiro–Wilk Test. For parameters without a normal distribution, the Wilcoxon rank-sum test was used instead. The dependence of the parameters on height, weight, and age was examined using a Spearman rank correlation.

**Results:**

Reference values for the various parameters were provided for an average population of healthy donkeys. For two parameters, heel length and sole thickness, a significant difference between the left and right limb was shown. There was evidence the medial wall angle was significantly bigger than the lateral wall angle (*p* = 0.0075), whereas the sole thickness was significantly larger on the lateral aspect (*p* < 0.001). No correlation with age was found for most variables, with a few exceptions. Almost all absolute lengths measured showed a moderate to strong positive correlation with the weight and height of the subject, while most angle measurements were not dependent on these parameters. The data obtained specifically for the donkeys in this study differ substantially from the data already published for horses. Thus, this data serves as a helpful guideline for the assessment of radiographs of the donkey’s foot. All donkeys included in this study were generally sound and lame-free in walk, but there was undeniable evidence of chronic laminitis and not all the values collected can be considered to be physiological.

## Introduction

It is generally accepted that the equine hoof is of utmost importance for the health and soundness of equids. The horn capsule protects internal structures, including soft and osseous tissues (1, 2). Orthopedic problems have a high priority in equine medicine and the foot is involved in the great majority of lameness cases (3). Trimming and shoeing are frequently performed processes that influence horses’ soundness to a great extent. Farriery, a very regularly used procedure, affects the hoof pastern axis and the shape of the hoof (4) and, subsequently, significantly influences horses’ soundness (1). A profound knowledge of normal conditions is the foundation for evaluating pathological findings. The foot is the most frequently radiographed anatomical region in a horse, and many studies have investigated horses’ normal radiological foot conformation by morphometric measurements (5–8), as they are an appropriate method to describe the foot conformation objectively (9, 10). It is commonly known that the foot conformation of horses and donkeys differ (11–16). Donkeys’ hooves are smaller in relation to their body, more boxy and steeper angled, and the sole is shaped like a U. The pastern axis of donkeys is much more upright. Hence, many authors doubt the unrestricted transferability of reference values, which had been investigated for horses (11, 15), and instead recommend that one should rather use reference values developed especially for donkeys (13). Nevertheless, only insufficient data exists describing the radiologic foot conformation in donkeys. Walker et al. (17) described the radiographic appearance of the feet of mammoth donkeys and the finding of subclinical laminitis. Collins et al. (11) examined the radiological anatomy of the normal and the laminitic donkey foot in lateromedial radiographs. El-Shafaey et al. (18) performed a morphometric evaluation of relevant radiographic parameters of clinically normal donkeys, and Mostafa et al. (14) did some morphometric measurements of the feet of working donkeys in Egypt. Most previous studies were restricted, with a small sample size or a very special population of subjects. Further studies have shown that hoof morphometry depends on individual aspects, breed, size, and body weight (19, 20). To the author’s knowledge, no study has performed morphometric measurements on lateromedial and dorsopalmar radiographs of generally sound donkeys’ forelimbs considering individuals of different sizes, breeds, genders, and ages. The present study aims to provide reference values for different radiographic parameters of the physiological and thus desirable hoof conformation of European donkeys’ front limbs to create guideline values and simplify the detection and quantification of deviations for farriers and veterinarians. Additionally, the authors aim to evaluate differences between the left and right limbs, just as between the medial and lateral parts of the hoof, as well as the dependence of various parameters on size, age, and weight.

## Materials and methods

### Donkeys

In this study, 46 donkeys of different ages, sex, and breed were included. The age of the donkeys ranged from three weeks to 40 years. Twenty-seven mares, ten geldings, and nine stallions were included in the study. The donkeys were presented in terms of this study by different scientific and private institutions, or they were inpatients in the Equine Clinic in Giessen due to non-orthopedic diseases. Non-orthopaedic diseases for which the donkeys were hospitalized in the equine clinic were mainly colic, removal of skin tumors and castrations. None of the subjects were known to have a metabolic disease that could affect hoof and horn quality, although this could not be completely ruled out in all cases. The donkeys were in a moderately thin to moderately fleshy nutritional state, the body condition score was 2 to 4 out of 5 (13). The most represented breeds were European domestic donkeys, including miniature breeds, the Poitou, Baroque donkeys, and mixed breeds. There were no special findings in the general examination, and the donkeys showed no lameness in walk. Exclusion criteria were lameness or current treatment with anti-inflammatory drugs as well as a reported history of lameness, especially due to laminitis. Most donkeys were barefoot, and the condition of their hooves required regular hoof correction by the farriers.

As very different individuals in terms of size, age, and weight were included in this study, the specimens were allocated in two different groups: the whole donkey population (Population A) and the adult, medium-weight domestic donkeys (Population B).

### Preparation

All relevant data, such as breed, age, sex, and living conditions of the donkeys, were collected. The animals were clinically examined and weighed before the radiographs were taken. Immediately before the radiological examination, all animals received a hoof preparation according to their fetlock status by one of the farriers, all of whom were educated in and are currently team members of the Clinic for Equine Surgery and Orthopedics of the Justus Liebig University in Giessen.

### Radiographic technique

**Figure 1:**
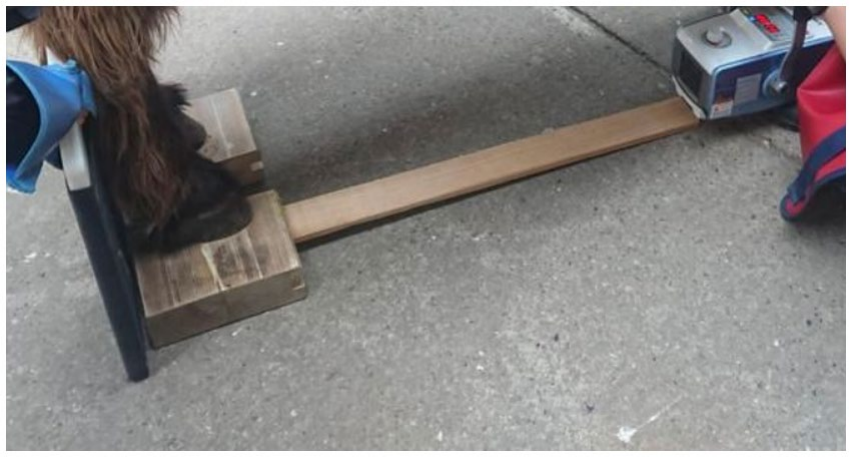
A standardized radiographic technique - the hooves were placed on a specially made wooden block, into which a wooden batten was inserted.

The radiological examination was performed according to a standardized procedure to obtain valuable results. Lateromedial (90°) and dorsopalmar (0°) radiographs of the distal toe of both forelimbs were taken as described elsewhere. The hooves were placed on the center of a specially made wooden block (23x18x8 cm), into which a 2 cm deep notch was milled cranially and laterally. The examiner took care that the donkey put equal weight on both front limbs, and the leg was positioned perpendicular to the ground. An 82 cm long wooden batten inserted into the notch always guaranteed the same film-focal distance (FFD) of 98 cm from the X-ray tube placed at the end of the wooden batten to the X-ray plate leaning against the wooden block. This method also made it easier to achieve a correct 90° projection. The setting can be seen in Figure 1. The settings of the X-ray equipment had to be varied due to the very different sizes and weights of the specimens.

### Radiopaque markers

Several radiopaque markers were attached to the hoof, as they are a very useful tool to optimize and simplify the performance of morphometric measurements (21). The coronary band was defined as the transition from hair to horn.

For the 90° radiograph, the dorsal hoof wall was marked with a wire centrally from the coronary band downwards. A small metal ball with a diameter of exactly 5 mm was attached to the center of the animal’s bulb on the palmar aspect of the coronary band. In addition to the exact marking of the heel, the metal ball also served as a reference size to calibrate length measurements. Furthermore, the sole was marked in the area of the coffin bone tip, respectively, the tip of the frog, by means of a paper clip applied longitudinally from the dorsal to the palmar aspect of the sole.

For the 0° radiograph, the paper clip was left in place. Additionally, the medial and lateral hoof walls were marked with a wire from the coronary band downwards at the broadest part of the hoof.

The X-ray images taken with a digital X-ray system were directly transferred and stored digitally for later evaluation.

### Morphometric measurements

**Figure 2:**
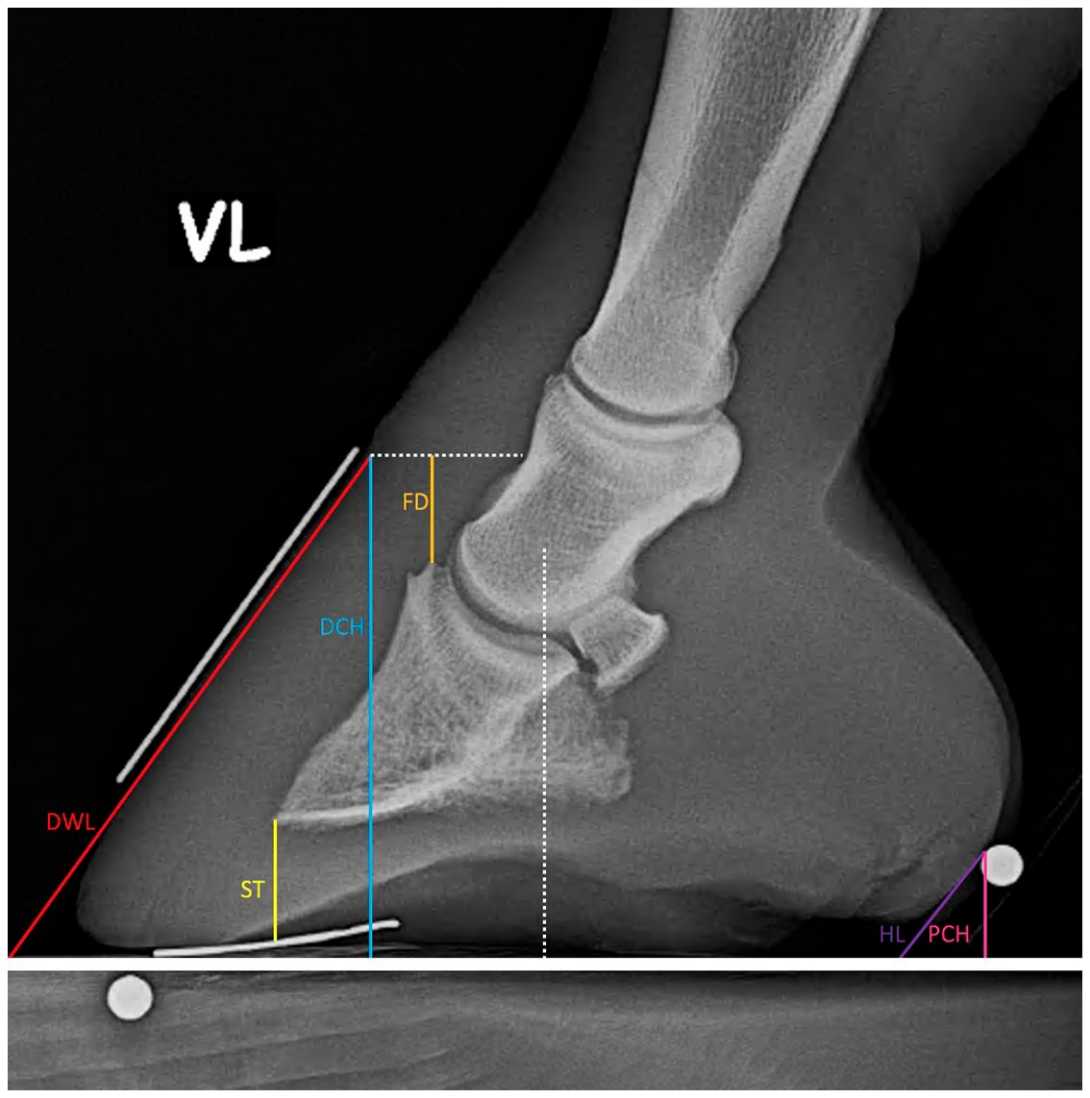
Length measurements on the lateromedial X-Ray.

**Figure 3:**
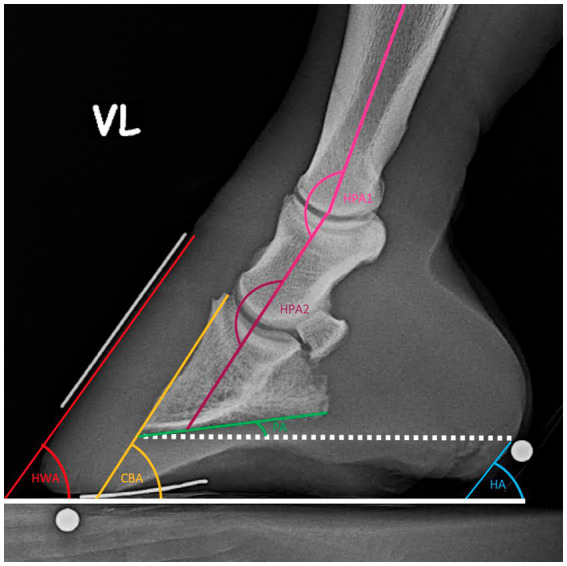
Angular measurements on the lateromedial X-Ray.

**Figure 4:**
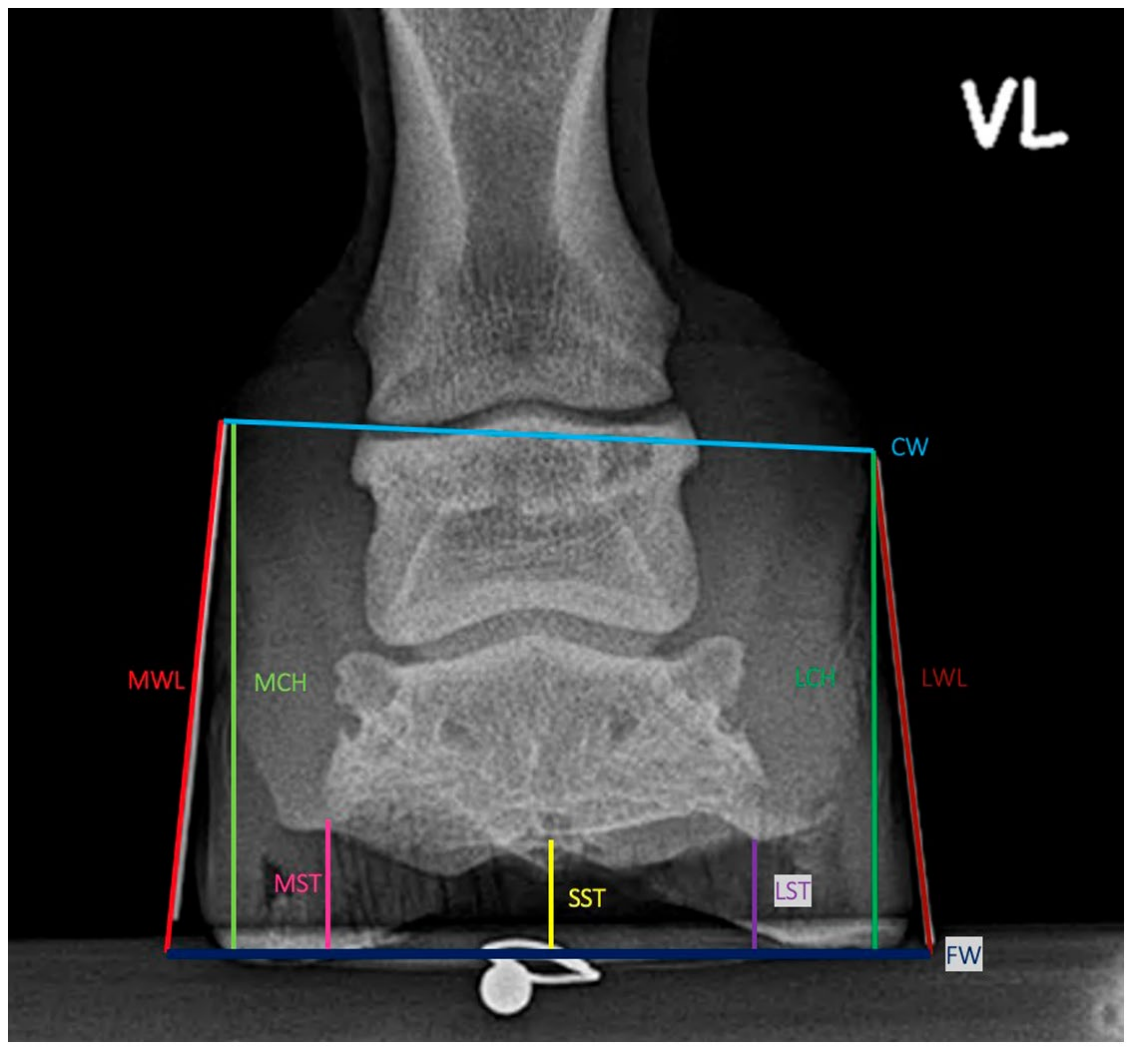
Length measurements on the dorsopalmar X-Ray.

**Figure 5:**
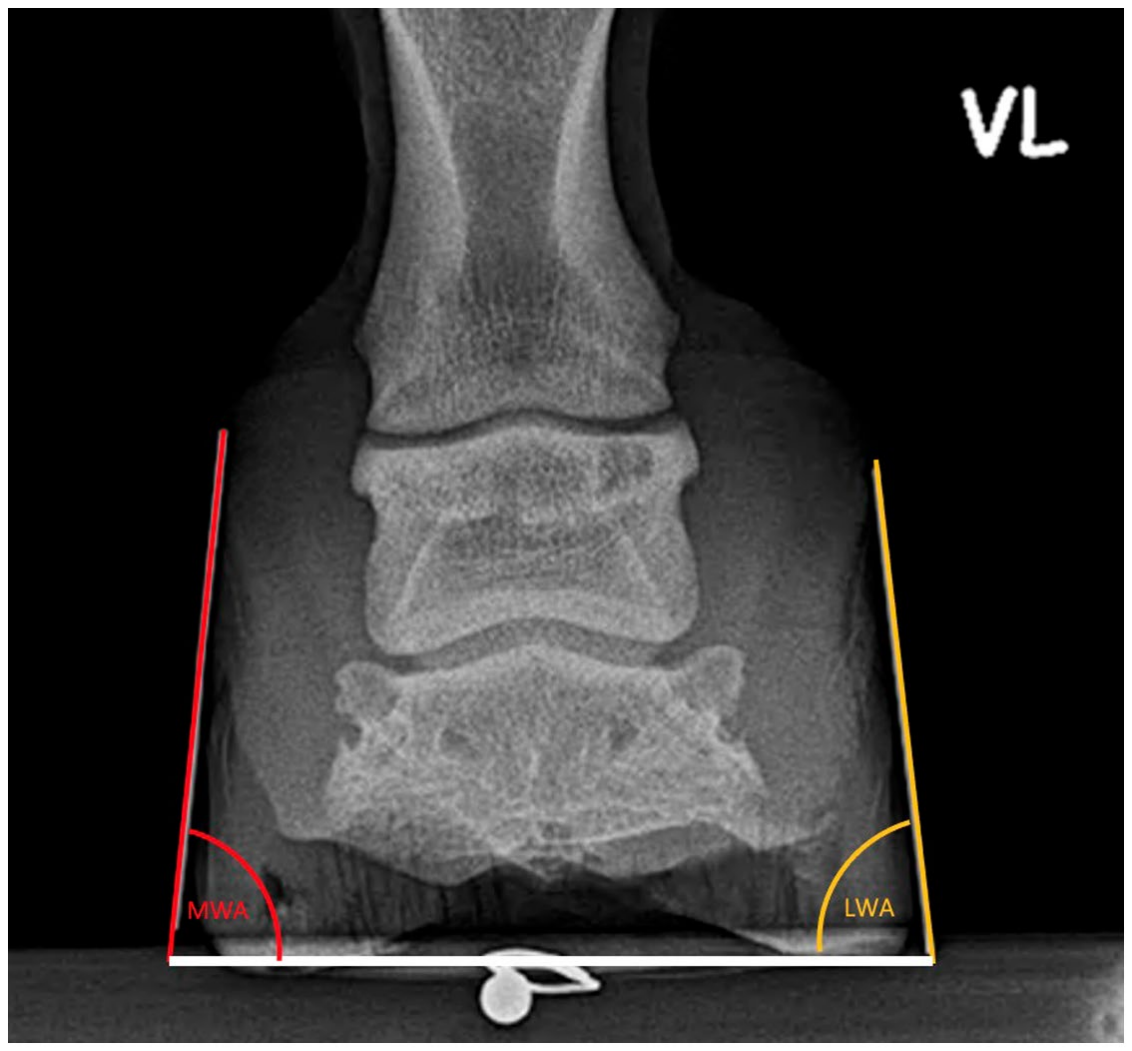
Angular measurements on the dorsopalmar X-Ray.

Morphometric measurements were obtained from both lateromedial and dorsopalmar radiographs. All the parameters taken are defined in Table 1 and visualized in Figures 2–5. The length measurements were taken as an original value and a standardized value by calibration to minimize the effects of magnification. Calibration was performed by dividing all length measurements by the measured diameter of the sphere and then multiplying them by the sphere’s real diameter of 5 mm.

**Table 1:** Definition of all measured morphometric parameters including full names and abbreviations.

Abbreviation	Measurement	Definition
Morphometric measurements on the lateromedial X-Ray		
Length measurements		
DWL	Dorsal hoof wall length	Distance from the proximal end of the wire at the coronet band along the dorsal hoof wall to the intersection of an imaginary extension of the dorsal hoof wall with the footing surface
ST	Sole thickness	Vertical distance between the coffin bone tip and the sole
DCH	Dorsal coronet heigth	Vertical distance between the coronet at the transition from hair to horn - marked by the proximal end of the radiopaque marker - to the sole
HL	Heel length	Distance measured from the attached metal ball in the region of the bulb to the palmar end of the sole along the contour of the heel
PCH	Palmar coronet heigth	Vertical distance between the metal ball attached to the heel and the footing surface
FD	Founder distance	Vertical distance from the level of the coronary band, which is marked by the proximal end of the radiopaque marker, to the extensory process of the coffin bone
Angles		
HWA	Hoof wall angle	Angle between the dorsal hoof wall and the footing surface (In case of an irregular dorsal contour of the hoof, the line was drawn according to the course of the radiopaque marker)
CBA	Coffin bone angle	Angle between the dorsal contour of the coffin bone and the footing surface (In case of an irregular dorsal coffin bone contour the line was drawn from the extensory process to the tip of the coffin bone)
CR_m / _c	Coffin bone rotation	Angle between the dorsal hoof wall and the dorsal contour of the coffin bone CR_m measured CR_c calculated = CBA - HWA
PA	Palmar angle	Angle between the palmar contour of the coffin bone and the sole
HA	Heel angle	Angle between the heel (connection between the bulb marked by the metal ball and the palmar end of the footing surface) and the sole
HPA1	Hoof Pastern Axis 1	Angle between the first and the second phalanx
HPA 2	Hoof Pastern Axis 2	Angle between the second and the third phalanx
Morphometric measurements on the dorsopalmar X-Ray		
Length measurements		
CW	Coronary band width	Distance between the proximal end of the lateral marker and the proximal end of the medial marker
FW	Width of the foot	Distance between the points where the medial and lateral hoof walls meet the ground
MWL	Medial wall length	Distance between the proximal and distal end of the marker of at the medial hoof wall
LWL	Lateral wall length	Distance between the proximal and distal end of the marker of at the lateral hoof wall
MCH	Medial coronet height	Vertical distance between the proximal end of the marker of at the medial hoof wall and the footing surface
LCH	Lateral coronet height	Vertical distance between the proximal end of the marker of at the lateral hoof wall and the footing surface
SST	Sagittal sole thickness	Vertical distance between the most distal point of the coffin bone and the footing surface at the half of the foot width
MST	Medial sole thickness	Vertical distance between the most distal point of the coffin bone and the footing surface at the most medial aspect of the coffin bone
LST	Lateral sole thickness	Vertical distance between the most distal point of the coffin bone and the footing surface at the most lateral aspect of the coffin bone
Angles		
MHA	Medial hoof wall angle	Angle between the medial hoof wall and the footing surface
LHA	Lateral hoof wall angle	Angle between the lateral hoof wall and the footing surface

### Statistical analysis

Firstly, a descriptive statistical analysis was performed with regard to the distribution of race, age, and weight of the donkeys. The mean value (x̄), standard deviation (x̄ +/− sd), minimum (min), and maximum (max) were determined for each parameter. For the length measurements on the lateromedial radiograph, the original values were compared with those standardized by calibration and tested for agreement. The CUSUM (Cumulative Sum) test for linearity and the Passing–Bablok regression were used for this purpose. The data was checked for a normal distribution using the Shapiro–Wilk test. In the case of a normal distribution of the data, a paired t-test was used to check for differences between the right and left limbs and between the medial and lateral parts of the hoof for various parameters. The Wilcoxon rank-sum test was used if the data were not normally distributed. In addition, a Spearman rank correlation was used to analyze the relationship between the individual parameters and age, weight, and size. All statistical tests were done for the whole donkey population (Population A) and the adult, medium-weight domestic donkeys (Population B). Additionally, all tests, except for the comparison between original and standardized values, were calculated for the original values and those standardized by calibration. For all tests, the significance level was set to *p* = 0.05. Statistical analysis was done in SAS® 9.4.

## Results

A total of 91 forelimbs of 46 donkeys were radiographically examined. In one foal the examination was discontinued after one leg due to the donkey’s extreme defensiveness. Most donkeys examined were European domestic donkeys (39 donkeys, 84.8%), followed by Poitou donkeys (five donkeys, 10.9%) and Baroque donkeys (two donkeys, 4.3%). The majority of the subjects (41 donkeys, 89.1%) were adults (3 years and older), and only five donkeys (10.9%) were juveniles (up to 2 years of age). On average, the donkeys were 11.86 years old. Twenty-five (54.35%) of the animals were assigned to the medium weight class (151–299 kg), 15 (32.61%) were small animals (up to 150 kg), and six donkeys (13.4%) were large (over 300 kg). The test subjects weighed 191.26 kg on average. The results of descriptive statistical analysis are shown in Table 2.

**Table 2:** Results of descriptive statistical analysis for right frontlimb (RF) and left frontlimb (LF) of the whole donkey population (Population A) and adult, middle-weight domestic donkeys (Population B).

Parameter		Limb	N	Mean (x̄) value	Standard deviation (x̄ +/− sd)	Minimum (min)	Maximum (max)
DWL	Population A	RF	45	81.41 mm	23.00 mm	43.39 mm	181.34 mm
		LF	46	80.41 mm	22.07 mm	40.56 mm	152.96 mm
	Population B	RF	24	74.03 mm	7.68 mm	52.42 mm	87.90 mm
		LF	24	74.45 mm	8.85 mm	60.11 mm	92.23 mm
ST	Population A	RF	45	16.66 mm	5.83 mm	7.95 mm	41.31 mm
		LF	46	15.82 mm	4.50 mm	7.71 mm	32.04 mm
	Population B	RF	24	15.48 mm	3.16 mm	7.95 mm	29.96 mm
		LF	24	14.52 mm	2.78 mm	7.71 mm	20.65 mm
DCH	Population A	RF	45	70.03 mm	19.82 mm	38.55 mm	159.46 mm
		LF	46	69.16 mm	19.11 mm	38.22 mm	133.43 mm
	Population B	RF	24	64.90 mm	6.84 mm	47.98 mm	78.20 mm
		LF	24	64.47 mm	8.82 mm	53.89 mm	86.12 mm
HL	Population A	RF	42	18.48 mm	5.65 mm	8.98 mm	32.39 mm
		LF	44	20.29 mm	5.95 mm	10.13 mm	36.11 mm
	Population B	RF	23	17.46 mm	5.21 mm	9.06 mm	29.23 mm
		LF	22	18.65 mm	5.17 mm	10.39 mm	28.37 mm
PCH	Population A	RF	42	14.92 mm	4.89 mm	7.16 mm	28.39 mm
		LF	44	16.28 mm	5.81 mm	7.70 mm	32.03 mm
	Population B	RF	23	14.35 mm	4.41 mm	7.73 mm	25.14 mm
		LF	22	14.91 mm	5.25 mm	7.70 mm	24.91 mm
FD	Population A	RF	45	18.39 mm	7.52 mm	5.57 mm	49.05 mm
		LF	46	18.50 mm	7.31 mm	6.49 mm	38.37 mm
	Population B	RF	24	15.89 mm	3.27 mm	5.57 mm	21.05 mm
		LF	24	16.69 mm	3.88 mm	10.24 mm	24.93 mm
HPA1	Population A	RF	45	9.27°	4.64°	−4.32°	16.67°
		LF	46	9.77°	4.01°	3.64°	22.29°
	Population B	RF	24	8.16°	4.94°	−4.32°	14.96°
		LF	24	8.91°	3.35°	3.64°	15.22°
HPA2	Population A	RF	45	−9.71°	10.26°	−28.40°	16.13°
		LF	46	−8.96°	8.71°	−25.91°	18.03°
	Population B	RF	24	−10.53°	9.21°	−24.42°	16.13°
		LF	24	−11.69°	7.20°	−25.91°	−0.44°
HWA	Population A	RF	45	58.28°	4.63°	46.77°	67.01°
		LF	46	57.97°	4.68°	48.45°	68.29°
	Population B	RF	24	58.85°	5.09°	46.77°	67.01°
		LF	24	59.10°	4.94°	48.45°	68.29°
CBA	Population A	RF	45	59.57°	4.62°	52.41°	70.72°
		LF	46	59.14°	4.47°	51.21°	69.76°
	Population B	RF	24	61.13°	4.81°	52.89°	70.72°
		LF	24	61.13°	4.48°	53.83°	69.76°
CR_c	Population A	RF	45	1.29°	3.65°	−4.92°	15.13°
		LF	46	1.17°	3.50°	−3.98°	15.39°
	Population B	RF	24	2.28°	3.63°	−3.56°	15.13°
		LF	24	2.03°	3.71°	−3.04°	15.39°
CR_m	Population A	RF	45	1.45°	3.73°	−4.21°	16.23°
		LF	46	1.27°	3.73°	−6.42°	16.28°
	Population B	RF	24	2.49°	3.80°	−3.33°	16.23°
		LF	24	2.32°	3.71°	−1.91°	16.28°
PA	Population A	RF	45	6.37°	3.98°	−1.30°	16.77°
		LF	46	5.43°	3.37°	−2.23°	13.35°
	Population B	RF	24	6.96°	4.23°	0.17°	16.77°
		LF	24	6.09°	3.23°	0.56°	13.35°
HA	Population A	RF	45	50.58°	8.82°	22.41°	67.30°
		LF	46	51.59°	8.82°	26.86°	77.69°
	Population B	RF	24	51.93°	8.96°	34.61°	67.30°
		LF	24	51.15°	8.26°	38.46°	69.15°
LWA	Population A	RF	45	87.47°	4.68°	79.67°	101.11°
		LF	46	86.87°	4.77°	76.06°	100.58°
	Population B	RF	24	85.89°	3.18°	79.69°	95.01°
		LF	24	85.76°	3.94°	76.06°	94.52°
MWA	Population A	RF	45	88.63°	4.88°	77.46°	101.16°
		LF	46	89.61°	5.72°	75.42°	99.92°
	Population B	RF	24	88.27°	3.97°	80.07°	95.12°
		LF	24	88.82°	5.16°	75.42°	98.28°
CW	Population A	RF	45	84.62 mm	23.87 mm	49.73 mm	190.19 mm
		LF	46	83.84 mm	24.82 mm	42.37 mm	187.29 mm
	Population B	RF	24	78.86 mm	9.89 mm	60.01 mm	110.56 mm
		LF	24	78.67 mm	10.67 mm	60.05 mm	102.68 mm
FW	Population A	RF	45	87.39 mm	22.36 mm	46.67 mm	176.71 mm
		LF	46	86.43 mm	23.20 mm	43.41 mm	174.98 mm
	Population B	RF	24	82.62 mm	10.47 mm	60.01 mm	110.59 mm
		LF	24	82.88 mm	11.24 mm	61.65 mm	115.20 mm
LWL	Population A	RF	45	62.96 mm	16.37 mm	32.38 mm	126.05 mm
		LF	46	63.20 mm	15.50 mm	29.43 mm	115.83 mm
	Population B	RF	24	60.88 mm	7.87 mm	46.69 mm	82.41 mm
		LF	24	61.15 mm	8.97 mm	48.88 mm	77.18 mm
MWL	Population A	RF	45	61.76 mm	16.94 mm	33.53 mm	136.28 mm
		LF	46	62.03 mm	15.61 mm	31.17 mm	112.46 mm
	Population B	RF	24	60.19 mm	9.59 mm	46.92 mm	94.39 mm
		LF	24	60.56 mm	9.62 mm	46.94 mm	84.50 mm
LCH	Population A	RF	45	62.42 mm	16.22 mm	33.79 mm	126.01 mm
		LF	46	62.36 mm	15.45 mm	29.17 mm	116.06 mm
	Population B	RF	24	60.24 mm	7.69 mm	46.13 mm	82.08 mm
		LF	24	60.83 mm	8.91 mm	47.70 mm	77.13 mm
MCH	Population A	RF	45	61.19 mm	16.96 mm	33.00 mm	134.87 mm
		LF	46	61.57 mm	15.25 mm	30.88 mm	110.55 mm
	Population B	RF	24	59.55 mm	9.68 mm	46.94 mm	94.40 mm
		LF	24	60.17 mm	9.46 mm	46.94 mm	84.09 mm
SST	Population A	RF	45	17.60 mm	5.30 mm	9.27 mm	32.17 mm
		LF	46	17.24 mm	4.99 mm	7.68 mm	35.93 mm
	Population B	RF	24	16.55 mm	4.07 mm	9.27 mm	24.58 mm
		LF	24	16.53 mm	3.59 mm	7.68 mm	23.57 mm
LST	Population A	RF	45	19.75 mm	5.73 mm	10.60 mm	38.15 mm
		LF	46	19.39 mm	5.59 mm	9.36 mm	38.69 mm
	Population B	RF	24	18.91 mm	4.49 mm	10.60 mm	29.78 mm
		LF	24	19.30 mm	4.42 mm	11.43 mm	28.08 mm
MST	Population A	RF	45	18.23 mm	5.60 mm	6.10 mm	33.13 mm
		LF	46	17.93 mm	4.89 mm	8.84 mm	37.31 mm
	Population B	RF	24	17.56 mm	4.22 mm	8.74 mm	25.92 mm
		LF	24	17.20 mm	3.26 mm	9.55 mm	24.11 mm

The abbreviations for the morphometric parameters used in the following section are defined in Table 1. A linear relationship between measured and calibrated values was demonstrated for all variables using the CUSUM test (*p* > 0.05). The Spearman rank correlation coefficient was r > 0.95 for all parameters and thus described a very strong positive correlation between originally measured and standardized values. The Passing–Bablok regression could neither prove a systemic nor a proportional difference between the two measured values for most parameters. Only HL (Heel length), PCH (Palmar coronet height), and FD (Founder distance) showed a proportional difference when evaluated for Population A. When looking at Population B, a proportional difference was proven for ST (Sole thickness).

The Shapiro–Wilk test and the QQ-Plot showed approximately a normal distribution for most variables’ data. Only ST and HPA2 (Hoof pastern axis 2) were not normally distributed when looking at Population A. After calibration, a normal distribution of data could not be proven for PCH.

A significant difference between the right and left limbs could only be proven for HL and ST (*p* < 0.05). The HL was significantly greater on the left than on the right when evaluating the original values of Population A. When looking at the original values of Population B or when using the standardized values, there was a significant difference in the ST between both front limbs, which was considerably greater on the right.

Comparing the medial and lateral parts of the hoof, a significant difference was shown for wall angle and sole thickness (*p* < 0.05). The MWA (Medial hoof wall angle) was significantly larger; therefore, the medial hoof wall is steeper than the lateral one. The sole thickness, on the other hand, was significantly larger on the lateral aspect of the hoof. No significant differences were observed between the medial and lateral wall length or coronet height.

For most variables, no significant correlation with the age of the test subjects could be proven (*p* > 0.05). When assessing the original and calibrated values of Population A, only CR (Coffin bone rotation) and FW (Width of the foot) showed a moderate positive and significant correlation with age. A weak to moderate positive and significant correlation could also be shown for CBA (Coffin bone angle), FD, LWA (Lateral hoof wall angle), LCH (Lateral coronet height), and CW (Coronary band width). In the evaluation of Population B, a moderate negative correlation was shown for HL, PCH, HWA (Hoof wall angle), and FW.

Numerous parameters, on the other hand, showed a significant correlation with the weight and size of the donkeys (*p* < 0.05). If there is a correlation with the weight, despite a few exceptions, the corresponding parameter correlates with the animal’s size, too. All length measurements on both radiographs (DWL (Dorsal hoof wall length), ST, DCH (Dorsal coronet height), HL, PCH, FD // CW, FW, LWL (Lateral hoof wall length), MWL (Medial hoof wall length), LCH, MCH (Medial coronet heigth), SST (Sagittal sole thickness), LST (Lateral sole thickness), MST (Medial sole thickness)) showed a moderate to very strong positive correlation with the weight and size of the test subjects, both when using the original measured values and when evaluating the standardized value. There was either no or a weak correlation (*p* > 0.05) of weight and size with all angle measurements on both projections (HWA, CBA, CR_c (Calculated coffin bone rotation), CR_m (Measured coffin bone rotation), PA (Palmar angle), HA (Heel angle), LWA, MWA, HPA2), with one exception. For the HPA 1 (Hoof pastern axis 1), which describes the axial deviation in the pastern joint, there was a moderate negative correlation shown with the size of the donkey (*p* < 0.05).

## Discussion

This study aimed to provide reference values for different radiographic parameters of the physiological and, thus, desirable hoof conformation of European donkeys’ front limbs. Additionally, the authors aimed to evaluate differences between the left and right limbs or between the medial and lateral parts of the hoof, as well as the dependence of various parameters on size, age, and weight.

The way to assess the hoof configuration most objectively and comparably is to perform radiographic measurements on high-quality x-rays. For example, concerning the hoof angle, there are different methods possible for the measurement of this parameter. Although you can achieve good and consistent results with measurements on digital photographs, too (22), measurement on correctly taken lateromedial radiographs is the most precise of all options (23, 24). With morphometric measurements on radiographs, the anatomical relationships between soft tissue structures, bones, and horn capsules can be precisely described (11). During the evaluation of measurements on X-ray images, one must always keep in mind that imaging a three-dimensional structure as a two-dimensional image presents certain challenges (25). Despite all efforts, the morphometric measurements on the X-ray image are still subject to a certain degree of error due to several aspects, such as imprecise limb positioning (2, 26) or inaccurate positioning of radiopaque markers (5), magnification effects, and false calibration (10, 27, 28), errors due to accidental obliquity (26, 28, 29), and the influence of farriery (4).

It has to be taken into account that there was a wide spread of values due to the very different individuals in the population in terms of size, age, and weight among the specimens of this study. To assess the population as a whole (Population A) and achieve a precise assessment of the most frequently represented type of donkey (Population B), all statistical tests were carried out and evaluated for both groups. The values of length measurements of extremely small and large animals, of course, differ clearly from those of the medium-sized subjects. Still, the average values in both groups are relatively similar except for a different standard deviation.

### Discussion of the results in comparison with the values of other authors

The results of the measurements in the present study compared to other authors’ results are shown in Table 3 for donkeys, where available, and horses. There is little specific data on morphometric measurements on donkey hooves in the literature, and, to the author’s knowledge, no reference values could be found at all for some morphometric parameters.

**Table 3:** Results of the present study in comparison with results of previous studies on donkeys, horses and ponies.

Parameter	Results of the present study (all donkeys, original values)		Results of other authors (Donkeys)		Results of other authors (Horses and Ponies)	
	Right frontlimb	Left frontlimb				
DWL	81.41 mm	80.41 mm	77.4 mm 67.3 mm	El-Shafaey et al. (18) and Mostafa et al. (14)	95–97 mm 77.8–78.0 mm	Kummer et al. (4) and Thieme et al. (8)
ST	16.66 mm	15.82 mm	24.3 mm	El-Shafaey et al. (18)	15 mm (pre-trimming) // 13 mm (post-trimming) 10.7 mm 10.6 mm	Kummer et al. (4), Masoudifard et al. (31), and Thieme et al. (8)
DCH	70.03 mm	69.16 mm	Not found		Not found	
HL	18.48 mm	20.29 mm	3.5 mm	Mostafa et al. (14)		
PCH	14.92 mm	16.28 mm	Not found		Not found	
FD	18.39 mm	18.50 mm	10.4 mm 25 mm	Collins et al. (11) and El-Shafaey et al. (18)	4.1 mm 11 mm 6.2 mm 9.4 mm	Cripps et al. (6), Kummer et al. (4), Masoudifard et al. (31), and Thieme et al. (8)
HWA	58.28°	57.92°	61.61° 70.2° 61.41° - 62.51°	Collins et al. (11), El-Shafaey et al. (18), and Khan et al. (44)	50.5° 54.2–54.8° 49.6° 54.01–54.37	Cripps et al. (6), Kummer et al. (4), Masoudifard et al. (31), and Thieme et al. (8)
CBA	59.57°	61.13°	61.11° 70.2°	Collins et al. (11) and El-Shafaey et al. (18)	49.4° 48.8–49.5° 48.5° 52.36–52.62°	Cripps et al. (6), Kummer et al. (4), Masoudifard et al. (31), and Thieme et al. (8)
CR	1.29°	2.28°	2.5°	Collins et al. (11)	−0.9° 0.4° −1.65-1.75°	Cripps et al. (6), Masoudifard et al. (31), and Thieme et al. (8)
PA	6.37°	5.43°	25.0°	El-Shafaey et al. (18)	<8° 5.4–6.4° 3.6° 6.02°	Cripps et al. (6), Kummer et al. (4), Masoudifard et al. (31), and Thieme et al. (8)
HA	50.36°	51.59°	54.71°	Mostafa et al. (14)	Not found	
HPA1	9.27°	9.77°	Not found		4.7–6.5°	Kummer et al. (4)
HPA 2	−10.2°	−8.96°	−4.3°	Collins et al. (11)	5.0–6.1° −0.2°	Kummer et al. (4) and Masoudifard et al. (31)
CW	84.62 mm	83.84 mm	Not found		85.06–85.02 mm	Thieme et al. (8)
FW	87.39 mm	86.43 mm	68.6 mm	El-Shafaey et al. (18)	104, 32–104, 42 mm	Thieme et al. (8)
MWL	61.76 mm	62.03 mm	55.3 mm	El-Shafaey et al. (18)	53, 05–53, 47 mm	Thieme et al. (8)
LWL	62.96 mm	63.20 mm	49.5 mm	El-Shafaey et al. (18)	53, 70–54, 41 mm	Thieme et al. (8)
MCH	60.74 mm	61.57 mm	Not found		52, 15–52, 68 mm	Thieme et al. (8)
LCH	62.42 mm	62.36 mm	Not found		52, 43–52, 86 mm	Thieme et al. (8)
SST	17.60 mm	17.24 mm	Not found		12, 78–13, 10 mm	Thieme et al. (8)
MST	18.23 mm	19.02 mm	Not found		16, 86–17, 01 mm	Thieme et al. (8)
LST	19.75 mm	19.39 mm	Not found		18, 70–19, 05 mm	Thieme et al. (8)
MWA	88.63°	89.39°	20.2	El-Shafaey et al. (18)	80.9–81.5°	Thieme et al. (8)
LWA	87.47°	86.87°	43.8	El-Shafaey et al. (18)	77, 36–78, 33°	Thieme et al. (8)

Comparing the values, especially angles, of different authors for horses and donkeys, it becomes clear that the data already differ considerably within the individual species.

### Dorsal wall length (DWL)

When comparing DWL to previous results of other authors it is absolutely necessary to differentiate whether hoof preparation was performed before the radiographic measurements were taken or not. The dorsal wall length is not an invariable hoof parameter but it is definitely influenced by hoof trimming, as it is reduced by 1.0–1.1 cm / 10% of the initial DWL throughout the trimming process (4). As for the DWL, it is noticeable that the average values of the present study are considerably larger than those of previous studies on donkeys. In the study by El-Shafaey et al. (18), which was performed pre-trimming and after removal of the shoes, the donkeys weighed an average of 150 kg, around 40 kg less than in the present study. The dependence of DWL on the size and weight of the animals has already been pointed out. In the study by Mostafa et al. (14), in which the examined donkeys were also not given a full trimming of their hooves prior to the radiological examination, the average weight of the test subjects was 186 kg, which was very similar to the population of the present study. However, the population of working donkeys kept in Egypt was clearly different and subjected to a different type and degree of stress. These two factors explain the difference between our results and those already available. Although in both previous studies measurements were performed pre-trimming, the results for the DWL were considerably smaller than the results for DWL in the present study. Added to that, in the study by El-Shafaey et al. (18) the donkeys were shoed and the shoes, which were removed immediately before the radiologic examination, should have protected the dorsal hoof wall against wear. If the effect of hoof trimming on the feet would be considered additionally and the DWL was reduced by about 10%, the difference between our measurements and the data published previously would be even more material. When comparing the values for DWL with those of horses and ponies, there is overwhelming agreement with the values from Thieme et al. (8). These authors examined morphometric parameters on ponies from 81.5–148.5 cm in height at withers in Germany post-trimming. Although no height was recorded for this population, a similar weight distribution and living and usage conditions to our donkey population can be assumed. Under these comparable conditions, the DWL of donkeys and ponies do not appear to differ in a noteworthy manner. In contrast, the study by Kummer et al. (4), which examined warmblood horses pre-and post-trimming, showed a 1.5–2 cm larger DWL than in the present study due to the huge difference in weight and size.

### Hoof angle (HA)

Probably the most frequently taken angle is the hoof angle. The hoof angle is important because it influences the forces on the superficial and the deep flexor tendon and the suspensory ligament (1, 4). It is out of debate that the donkey has a steeper hoof wall angle than the horse (11, 12, 17, 18). This fact was confirmed in our data set and should, therefore, not be discussed further.

### Coffin bone angle and coffin bone rotation (CBA and CR_c / CR_m)

It is generally accepted that the dorsal hoof wall and the dorsal contour of the coffin bone of equids should be straight and nearly parallel to each other (5, 21), which means the CBA should be equivalent to the HWA (30). Previously published values for the CBA in donkeys are very similar to our findings. Due to the parallelism between the dorsal contour of the hoof capsule and the dorsal contour of the coffin bone, the CR, which is defined as the divergence between HWA and CBA (11), should go toward zero. For horses as well as for donkeys, it is reported that there can be a slight difference between HWA and CBA (4, 5, 7, 21, 23, 31). Dependent on the study a CR of up to 2° (11) to 4° (32) or 5° (33) is not considered to be clinically relevant. The mean CR in the present study was 1.19°–2.28° and, therefore, is definitely within the normal range for horses and similar to a previously described value for donkeys (11). Nevertheless, when looking at our results in Table 2, it becomes clear, values were recorded for coffin bone rotation in generally sound donkeys that certainly cannot be classified as physiological. All the animals included in our study were without special findings in the clinical examination, lame-free in walk and the owners were not aware of any previous laminitis. Nevertheless, some of the animals showed considerable changes in position of as well as structural changes in the coffin bone.

### Palmar angle (PA)

One parameter that has increasingly come into focus in recent studies is the palmar angle. The specifications for a normal PA differ clearly in the literature, and the information is within the range between 2°–10°. Redden (3) claims the PA is correlating positively with the HWA (3). The mean PA in our study was 5.43°–6.37°, which matches previous findings. The PA of a horse and a donkey seem to be very similar (13). A very small or negative PA can result in increased stress on several soft tissues, such as the deep digital flexor tendon, and cause chronic foot pain and lameness (21). In contrast, a large PA is suspicious for the presence of a coffin bone rotation and can be associated with pathologies like a club foot or laminitis (34).

### Heel angle (HA)

The heel angle should be nearly the same or not more than 5°–10° smaller compared to the HWA (21), otherwise, the equid was suffering from so-called underrun heels. In this study, the HA is, on average, less than 10° smaller than the HWA and is within the normal range described for horses. One aspect that could lead to the slight difference between HWA and HA in the majority of equids could be the influence of trimming and farriery (23). The influence of the previous hoof preparation on this measurement must also be considered in the present study.

### Sole thickness (ST)

A sufficient sole thickness is necessary to protect osseous and soft tissues within the horn capsule (19). A small ST can also be a hint of a dislocation of the coffin bone (35). For horses, the mean ST is 11 mm (5). Redden (3) suggests the minimum ST should be 15 mm; otherwise, the sole will have a disrupted perfusion. This will result in smaller and lower horn growth in the area of the sole. The ST in the present study appears to be the same or slightly greater than in horses and ponies in both lateromedial and dorsopalmar radiographs. However, our results differ from the data published by El-Shafaey et al. (18) who found considerably greater sole thicknesses in donkeys in Egypt. This is possibly due to a heavier workload of the animals on dry and hard soils and, as a result, heavier abrasion of the hoof horn. Once again, the aspect of hoof processing must be considered. The sole thickness of warmblood horses was reduced by an average of 2 mm (13%) as a result of hoof trimming (4). The difference between the data previously published and the values determined in the present study is considerably greater, even if 13% of thickness was substracted from the values determined by El-Shafaey et al. (18). Nevertheless, according to Kummer et al. (4) the sole thickness stays relatively constant throughout the trimming process as the net growth and the natural wear seem to be almost equal. Subsequently, the influence of trimming to this measurement can nearly be neglected, as only loose horn is removed during hoof processing.

### Founder distance (FD)

A radiometric parameter often considered when assessing X-rays of the equine foot regarding chronic laminitis is the founder distance. Several authors claimed that the coffin bone was located more distally in a donkey than in a horse (12, 13, 18) and, therefore, a donkey had a bigger FD in relationship to their body size. Hence, the donkey’s extensor process of the coffin bone is not in line with its coronary band (12). Collins et al. (11) could already confirm this empirical conception as they evaluated FD-values three times as big as in a pony of similar size. The mean FD in donkeys published in previous studies is between 10 mm (11, 13) and 25 mm (18). In conclusion, the founder distance seems to be greater and less expressive in donkeys than in horses (18).

### Hoof pastern axis and the club foot conformation in donkeys (HPA1 and HPA2)

Axial alignment of all phalanges should be considered normal in equids (19, 30, 33) and provides optimal mechanical conditions (4). Nevertheless, a pastern axis broken backwards in the pastern joint and broken forwards in the coffin joint was frequently found in the donkey population in a previous (14) as well as in the present study. Hence, some authors claim that in donkeys, a slightly broken forward pastern axis can also be accepted as a normal finding (12–16). These facts lead to the discussion about whether a club foot conformation is a normal finding in donkeys. In horses, the club foot is defined as a blunt angled hoof with a hoof angle of more than 60° and high heels combined with a broken forward pastern axis (36). This foot conformation is predisposing for heels-first-landing, results in increased forces on the heel, and can, therefore, lead to inflammations of the coffin joint and sole bruising (1). The limit for the dorsal hoof wall angle in horses with club foot conformation is 60°, approximately 5°–15° above the physiological front wall angle of 45°–55° described for the horse (37). Since the donkey has a 5°–10° steeper hoof wall than the horse, this would mean a dorsal hoof wall angle of at least 70° with a toe axis broken forward at the coffin joint if the criteria for a club foot were applied to the donkey. All these criteria combined could be found very rarely in the population of the present study. Consistent with the opinion of other authors (38), all these aspects lead to the conclusion that club foot conformation should not be considered normal in donkeys. A straight pastern axis should be aimed for. The relatively frequent occurrence of a broken toe axis can most probably be explained by the fact that many donkeys receive hoof preparation not at appropriate intervals. However, correcting a broken toe axis requires not just a one-off treatment but consistent and regular trimming of the hooves over a longer period.

### Parameters with relevance to dorsopalmar foot conformation

#### CW, FW, MWA, LWA, MWL, LWL, MCH, and LCH

While the coronet width CW found in the present study is very similar to the one published by Thieme et al. (8), the FW is much smaller. This can be explained simply by the fact that donkeys have not only a steeper dorsal but also up to 10° steeper medial and lateral hoof wall than horses and ponies (16). The medial and lateral coronet height, as well as the wall length, seem to be bigger than in the ponies examined by Thieme et al. (8).

### Comparison between the medial and lateral parts of the hoof

When assessing the measurements on the dorsopalmar X-ray and therefore evaluating the mediolateral foot balance and symmetry, the examiner should take into account the high importance of correct positioning of the limb (9, 21). A bilateral symmetric foot conformation is considered normal (21), which means a line bisecting the third metacarpal bone perpendicular to the ground should also bisect the foot (21). The medial and lateral hoof wall length should be similar, and the medial and lateral hoof wall angle should be nearly the same and should both be below 90° so the sole is broader than the coronary band (35). One has to remember that in donkeys, not only the dorsal but also the lateral and medial hoof walls are steeper than in horses (16).

Former studies have shown mediolateral imbalance is common in horses and seems to be normal to a certain extent (39). Mediolateral imbalance is present if the measurements of medial and lateral hoof wall length differ more than 5 mm (14). This can result in unequal force application and, consequently, in sheared heels, cracks, or lameness.

Former studies on horses have shown that a larger lateral part of the hoof combined with a steeper medial hoof angle is common (9, 21). This matches the results of the present study, as a significantly steeper medial hoof wall angle was also observed. The hoof shape should not be assessed separately but always in context with the position of the limbs. Just as in horses, a regular limb position is optimal in donkeys but is very rare in practice. In the subjects of the present study, a slightly toe-width limb position was observed in many donkeys, resulting in a significantly steeper medial hoof wall angle.

Although the medial and lateral sole thickness should be approximately the same, about 63% of all horses show a significantly lower medial than lateral sole thickness (21). Also, in chronic founders, it can be seen that the sinking of the coffin bone is not always equal but often more severe on the medial aspect, causing a smaller medial sole thickness. This finding can be fully supported after considering the present study’s data, as a significantly lower sole thickness on the medial aspect of the hoof was also shown.

### Correlation with the body weight

Further studies have shown a correlation between different hoof parameters and the hoof size to the body weight (14, 38). The hoof has to be big enough to carry the body weight adequately (38). If the hoof is too small in relation to the body size, the horn capsule cannot protect the soft and osseous tissues strongly enough (38). Turner (9) claimed a hoof is too small if it has to carry more than 5.5 kg of weight per m^3^. Especially concerning the DWL, a significant correlation with the body was shown in previous studies (9, 15, 19). In the present study, the dispersion of results was very high for this value, and DWL depended on the donkey’s body weight, too.

The current study showed a moderate to strong positive correlation not only of the DWL but of all linear parameters with the height and weight of the test subjects in both X-ray projections. There was a somewhat weaker but still significant and detectable correlation with the sole thickness in both projections and with FD when only Population B was considered. The finding that FD is not dependent on the size of the animal to the same extent as the rest of the linear parameters has already been detected by other authors before (4, 7). In contrast, there was no significant correlation with most of the angular measurements. Only HWA and CBA correlated minimally positively with height and weight. Larger donkeys, therefore, appear to have a slightly steeper hoof than smaller donkeys. On the other hand, there was a weak to moderate negative correlation with HPA 1. Consequently, heavier donkeys seem to be less prone to a toe axis that is broken backwards in the pastern joint than smaller donkeys do.

### Correlation with the age

No correlation with age was found for most parameters. Only the CR correlated moderately positively with the age of the test subjects. This is accompanied by a weak correlation of CBA, which automatically becomes steeper during rotation. In addition, a weak correlation of FD with age was discovered. The correlation of all these parameters with the age of the animal seems logical because a significant CR is often seen and considered to be pathognomonic in horses with chronic laminitis (21, 30, 40) due to the loss of function of the suspensory apparatus of the coffin bone (21, 41). An older animal naturally has a higher probability of suffering from laminitis during its longer life, which causes such a change in the position of the coffin bone.

In the dorsopalmar X-ray, some parameters also correlated weakly but significantly with the subjects’ age. There was a positive correlation with the FW and CW, whereas there was a negative correlation with LWA. Therefore, the hoof appears to tend to become wider and laterally less steep with age.

When looking only at Population B, there was also a moderate negative correlation between age and the HWA, HL, and PCH. Therefore, the hoof appears to become somewhat flatter, and the heels slightly lower with age.

In summary, with regard to the age dependency of various parameters, it can be stated that the hoof shape changes marginally differently with increasing age, and typical radiographic changes caused by chronic laminitis can be found comparatively more frequently. Although only clinically healthy animals were included in the study, some animals showed radiographic changes in the sense of chronic laminitis. Due to a donkey’s high pain tolerance (42), donkeys with laminitis show very different clinical signs compared to horses with this disease, and therefore, acute and chronic laminitis are often not detected in early and mild stages (13, 16). In donkeys, clinical symptoms of laminitis often do not appear until displacement of and degenerative changes in the osseous structures are already evident (43). Hence, it seems likely some donkeys with mild chronic laminitis that never caused perceptible clinical signs also were included in the present study and had some impact on the parameters that were measured.

### Limitations of the study

The present study is limited by a clinically extensive and versatile but statistically comparatively small sample size. The specimens used in the study (Population A) are very different in age, weight, and size and thus are only restrictedly comparable. Subsequently, an even smaller sample size was examined in a second step (Population B) to have subjects more similar and comparable and reduce standard deviation. Furthermore, the fact that different farriers prepared the donkeys’ feet may have further influenced the data, although all of the handling farriers underwent an equal education in the Clinic for Equine Surgery and Orthopedics of the Justus-Liebig-University Giessen and followed an equal and accepted technique of hoof trimming. In addition, two different examiners took the X-rays, which might have also influenced the results.

## Conclusion

All in all, it is not up for discussion that there are substantial differences between a horse’s and a donkey’s hoof. This paper provides useful reference values for several morphometric measurements of the hoof of normal donkeys as a guide for trimming and evaluating changes in foot conformation by farriers and veterinarians. It is necessary to be aware of the fact not every donkey that appears lamefree really is basically free of orthopedic disease or pathological changes of the toe. Donkeys and horses differ not only concerning anatomy, but also in terms of their interior and pain expression. Foot problems are the main cause of lameness in donkeys and are often associated with deviating values for morphometric measurements. Although all donkeys included in this study were generally sound and lame-free in walk, there was undeniable evidence of chronic laminitis in several of the specimens. Consequently, it is of tremendous importance not to consider the measurements performed in isolation but always in connection with each other and the clinical picture.

## Data Availability

The raw data supporting the conclusions of this article will be made available by the authors, without undue reservation.
